# Supplementary material for: Thermal Hall conductivity in the cuprate Mott insulators Nd2CuO4 and Sr2CuO2Cl2
Source: Nat Commun. 2020 Oct 21;11:5325. doi: 10.1038/s41467-020-18881-z (PMC7577976; doi:10.1038/s41467-020-18881-z)
Supplement: Supplementary file 1 — Supplementary Information [file 41467_2020_18881_MOESM1_ESM.pdf]

Thermal Hall conductivity in the cuprate Mott insulators Nd_2CuO_4 and $Sr_2CuO_2Cl_2$

Supplementary information

Marie-Eve Boulanger[1,*], Gaël Grissonnanche[1,*], Sven Badoux[1], Andréanne Allaire[1], Étienne Lefrançois[1], Anaëlle Legros[1,2], Adrien Gourgout[1], Maxime Dion[1], C. H. Wang[3], X. H. Chen[3], R. Liang[4], W. N. Hardy[4], D. A Bonn[4] and Louis Taillefer[1,5]

1 Institut Quantique, Département de physique & RQMP, Université de Sherbrooke, Sherbrooke, Québec J1K 2R1, Canada

2 SPEC, CEA, CNRS-UMR3680, Université Paris-Saclay, Gif-sur-Yvette, France

3 Hefei National Laboratory for physical Science at Microscale and Department of Physics, University of Science and Technology of China, Hefei, Anhui 230026, People's Republic of China

4 Department of Physics & Astronomy, University of British Columbia, Vancouver, British Columbia V6T 1Z1, Canada

5 Canadian Institute for Advanced Research, Toronto, Ontario M5G 1M1, Canada

* *contributed equally to this work.*

Supplementary Figure 1 | Thermal Hall conductivity measurement

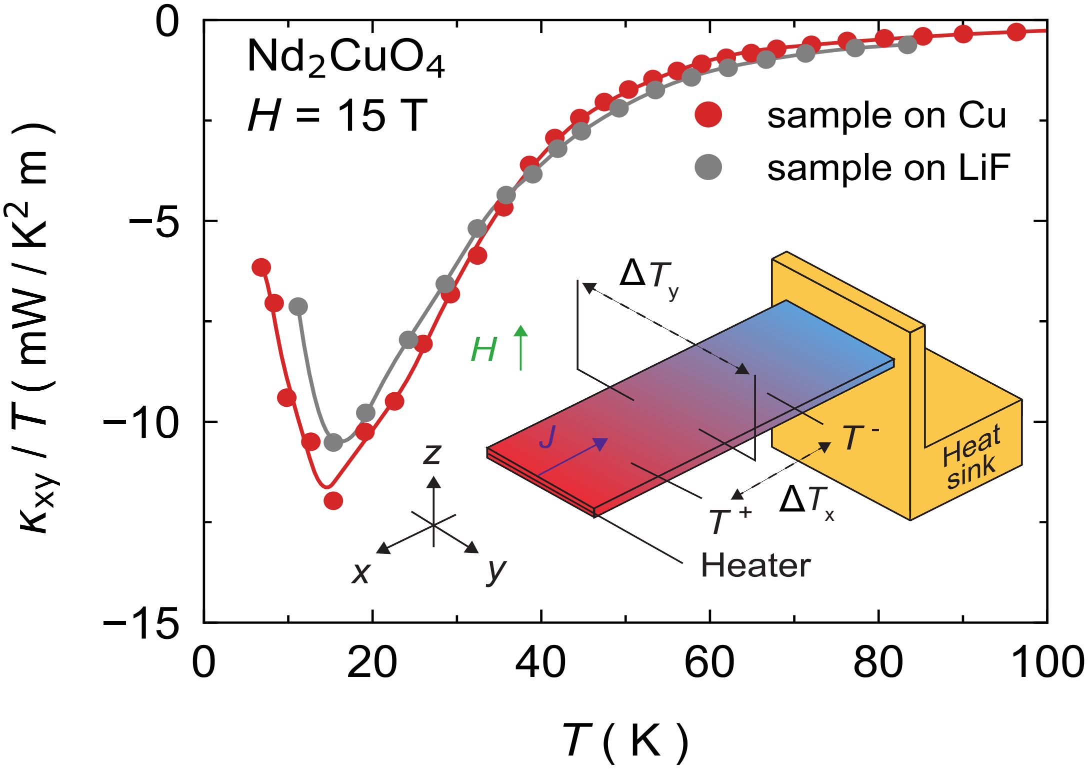

Thermal Hall conductivity κ_{xy} of our Nd_2CuO_4 sample, measured in a magnetic field H = 15 T, plotted as κ_{xy}/T vs T. Two measurements were carried out: one with the heat sink made of copper (red data points) and the other one with the heat sink made of LiF (gray data points). Inset: Sketch of the measurement setup. One end of the thin sample is glued to a heat sink, while the other end is heated using a resistive heater attached to the sample by a silver wire. The heat current J generates a longitudinal temperature difference ΔT_x, both along the length of the sample (x direction). A magnetic field H applied along the z direction produces a transverse temperature difference ΔT_y between the two sides of the sample, along the y direction.
